# Incidents of the Hospital Meetings

**Published:** 1887-06-25

**Authors:** 


### Incidents of the Hospitals Meetings.

By One who Went to them All.

**A Converted Listener.**

"Well, I don't see as the hospitals 'as much to do with me," soliloquised an individual as he spelled out a bill before the Shoreditch Town Hall; "them big buildings 'as got plenty of money without asking us poor chaps, so I ain't agoin' to give nothink; but I see as there ain't no collection agoin' to be made, and I'd like to see that 'ere Marquis of Lawn, so I'll go in and look roun' about a bit." I watched this interesting personage take a seat in the dingy hall—the scene of many a fierce political fight. His personal appearance was by no means of the comeliest type; he had evidently come direct from his toil. Dirt-begrimed and unkempt as he was, he boldly essayed to take a seat in the second row, but his personal attire was of such a character that I particularly noticed that incomers, if they did not exactly give him a wide berth, at least kept a chair or two off. I noticed further that he listened with an evident interest to the amusing bits of medical folk-lore with which the Bishop of Bedford "padded" his introductory speech. Then the persuasive appeal of Dr. Walsham How seemed to exert its influence upon his untutored mind, and finally, when the "Marquis of Lawn" spoke, I observed that our friend was leaning forwards with his hand to his right ear, evidently bent on understanding all he could. After the meeting was over I ventured to speak to this outcast specimen of humanity, offering him by way of introduction a copy of The Hospital. He thanked me, and said with a smile, "I think, sir, as this was the paper as I seed the chairman a-reading." I assented, for I certainly did see the Bishop of Bedford perusing The Hospital during the proceedings. "I'm agoin', sir," he continued, "to give a bit of silver on Sunday to the Hospital Sunday Fund, and do you know why, sir?" I ventured to suggest that probably the arguments he had heard had convinced him. "No, sir," he said, "I don't know much about argiments, but I 'eard as what Mr. Cuff said, and I knows as what Mr. Cuff says must be right, so I'm goin', as I said, to give a bit of silver on Hospital Sunday." The name of the Rev. W. Cuff is, I may explain, a household word in Shoreditch, a fact which his hearty reception at the meeting in question must have made amply manifest to all.

**An Incident at the Lambeth Meeting.**

In attending the whole of the meetings held in support of the Hospital Sunday Fund, I have, I believe, accomplished a unique task. Like dear old Samuel Pepys, I never go abroad without making a "mem" of anything that strikes me. "When found make a note of," says Captain Cuttle, and I certainly think some of us would be wiser if we did sometimes make a note of what we hear. The public, as a rule, are too apt to forget on the Tuesday what they heard on the Monday; and, as far as regards the hospitals, they do not always trouble to hear what is said on the Monday. The first meeting, which was held by the kind permission of the Archbishop of Canterbury at Old Lambeth Palace, just bears out my point. I scanned down the list of signatures, and almost without exception I found the autographs were those of persons whom I know are staunch supporters of the hospitals of London. It is not much use to carry coals to Newcastle, and hence I do not think that the eloquence of the Rev. Newman Hall and the other speakers told to much account. The people whom the friends of the Hospital Sunday Fund want to get at are the "outsiders"; and if the "outsiders" will not come to hear, it might perhaps be worth while to imitate the philosophic decision of Mahomet, who when he found the mountain would not come to him, straightway resolved to go to the mountain.

The proceedings at the Lambeth Palace meeting were enlivened by an incident occasioned by some remarks of the Rev. Mr. Lee, a clergyman of great talent, great energy, and great benevolence. I fully believe that the Hospital Sunday Fund has no better well-wisher than Mr. Lee, but somehow Mr. Lee has a knack of raising issues which are ill-timed and ill-advised. At the Archbishop's meeting, for example, Mr. Lee tried his best to bring a charge of extravagance against the local Lying-in Hospital, forgetful of the fact that the meeting was called, as Dr. Benson more than once explained, for specific purposes, namely, to advocate the claims of the hospitals in general, and not for investigating the expenditure of any one particular institution. The *Dispatch* of last Sunday said, " The rev. gentleman was promptly ruled out of order by the Archbishop." What I wondered at was that the Archbishop was so forbearing as to allow Mr. Lee to waste so much of the time of the meeting as he did. As Mr. Burdett pointed out, if he has a case against the institution in question, he should lay his evidence before the Mansion House Committee.

"Fads."

The Hon. Henry Noel treated the Paddingtonians to a disquisition on the subject of etymology. In attempting to be practical by offering a suggestion as to how the number of subscribers to our hospitals might be increased, Mr. Noel went out of his way to proclaim himself a " faddist," and to go into the derivation of the term, venturing—very unfortunately for his philological reputation—to connect "fad" with the old French word *fadaise*, foolery, and instanced, as in alliance with these, the word " fade." It may interest Mr. Noel to know that we have not got our " fad" from the French, but from the Anglo-Saxon verb *fadian*, to arrange; while I may add that the etymology of the word "fade" is altogether unknown. But what has the question of the etymology of the word " fad" to do with the much more pressing question of raising money for the hospitals? I wondered, too, but Mr. Noel eventually unfolded his "fad." "Those of you," he said, " who insure your lives know that the sums paid for premiums of assurance may be deducted from the amount of income chargeable with income-tax. The principle the Government goes on is, that if a man insures his life he shall pay less income-tax for so doing. Now, my fad is this, that everyone who is a subscriber to a hospital shall have the right to deduct from his poor-rate what he subscribes to the hospital, always supposing that it does not exceed a third." Mr. Noel's suggestion—" fad," I should say—is certainly worthy of attention, for doubtless, if it could be carried into practice, thousands of cases, now charged upon the rates, would be relieved by the voluntary efforts of the people who subscribed to the hospitals, while it would urge all to liberality and charity.

Practical Suggestions.

A man, we know, is nothing if he is not practical, and hence I did anticipate that more of the great speakers who rallied to the support of the Hospital Sunday movement would have offered suggestions for making the Fund the Fund of the people, instead of the Fund of the church and chapel-goers alone. Everybody urged the grand work which the hospitals are doing for suffering humanity; everybody pointed out why, looking at the hospitals from a purely personal and selfish point of view, we should support them because they are the training ground of the coming generation of doctors, and produce trained nurses ever ready to enter our sick homes; everybody urged the nobility of charity; how the healing of the sick has been associated with the Christian Church from the earliest times; how, as a community, it was incumbent upon us to discharge our duties and responsibilities; but, while the speakers pleaded to their audiences to give liberally on Hospital Sunday, and to influence their friends to do so too, practical suggestions for the extension of what I may call the "area of operations" were few and far between. The problem is, I know, a very difficult one, but, in view of the crisis which threatens so many of our hospitals, it is imperative that we should attempt its solution. Probably not a tithe of our adult population go to places of worship on Sunday. It is these " un-get-at-able" people, as one speaker phrased it, whom we want to convert into friends and supporters of our hospitals. By far the best suggestion, tending in this direction, was that made at the Kensington meeting by the Duke of Argyll. Sir Algernon Borthwick promised to see if he could put the necessary machinery in motion, and I hope soon to hear that the experiment is being tried. The Vicar of Kensington proposed a very " thorough" process for alms-collecting, but I fear it would be wanting in practicability. He would take the subscription and donation lists of all the metropolitan hospitals, and evolve from them one complete list of names. This he would print and place in the hands of a staff of voluntary collectors in each district, who would visit the houses of all non-donors or non-subscribers, and attempt by a little moral suasion their conversion. Mr. Pike (formerly secretary to the St. Mark's Hospital for Fistula) made two or three suggestions at the Lambeth meeting, the most ingenious of which was, perhaps, that lawyers, in preparing wills for their clients, should impress upon them the importance of not forgetting the hospitals.

One Unbeliever.

The whole of the meetings called in support of the Sunday Fund movement revealed only one unbeliever—at least, only one who had the courage of his convictions. At the Shoreditch meeting, just as the Bishop of Bedford was about to put the resolution, a person rose in the gallery to oppose the motion; he did not believe in hospitals as " Christian institutions"; he did not like to go there and be " under the inspection of the doctors"; while he believed the Hospital Sunday Fund tended to make the poor dependent on the rich. Such arrant nonsense as this shows how essential it is that the masses should know more about hospitals than they do, and how important it is they should be shown their error in regarding them as institutions supported solely by the rich for the use solely of the poor. The opponent of the resolution evidently considered the hospital as a kind of adjunct to the parish union, and consequently saw in the Sunday Fund an instrument which was assisting to pauperise the people by making them dependent for medical relief upon the wealthy. When all classes recognise the duty of contributing, according to their means, the fallacious notion about " pauperising" will die out—then, but not before.

State Support.

The question of the intervention of the Government, if our hospitals failed to find continued adequate support, was brought forward at all the meetings. With two exceptions only, the speakers were unanimously in favour of the present system of voluntary support. Lord Rosebery devoted the greater portion of the splendid speech he delivered at the Stratford meeting to the consideration of this question, and his powerful advocacy of the voluntary system can leave little doubt that it is the best. Dealing with this subject at the Kensington meeting, Mr. Brudenell Carter remarked, that we ought not to forget that the London hospitals were used by large numbers of people who were not Londoners, but who came from all parts of the British Isles, adding that one-half of his patients at St. George's were persons from the provinces.

"The Hospital."

A noticeable incident at the meetings was the sale of THE HOSPITAL, a very considerable number of copies being disposed of by the ladies and gentlemen who kindly undertook to attend the meetings for this purpose. The object of the proprietors of this Journal was twofold, namely, to assist in spreading knowledge about our hospitals, and, secondly, to practically help the Hospital Sunday Fund, the whole of the profits on the sale of the papers at these meetings being devoted to that purpose.

